# A Pilot Study Evaluating the Effects of Early Intervention for Italian Siblings of Children with Autism Spectrum Disorder

**DOI:** 10.3390/brainsci11111381

**Published:** 2021-10-21

**Authors:** Valentina Riva, Elena Maria Riboldi, Barbara Urbani, Massimo Molteni, Laura Villa

**Affiliations:** Child Psychopathology Unit, Scientific Institute, IRCCS Eugenio Medea, Bosisio Parini, 23842 Lecco, Italy; elenamaria.riboldi@lanostrafamiglia.it (E.M.R.); barbara.urbani@lanostrafamiglia.it (B.U.); massimo.molteni@lanostrafamiglia.it (M.M.); laura.villa@lanostrafamiglia.it (L.V.)

**Keywords:** autism spectrum disorder, early intervention, siblings, imitation, joint attention

## Abstract

Autism spectrum disorder (ASD) is a high-cost/high-burden problem. Early intervention may prevent development of the disorder, improving child outcomes and reducing long-term consequences. However, few studies have investigated the role of early intervention in children younger than two years. This study aims to examine the effect of early intervention in 18-month-old high-risk siblings of children with ASD (HR-ASD) with clinical signs of autism. The intervention is based on the principles of Applied Behavior Analysis and focuses on the development of early precursors to social and communicative competence (joint attention and imitation behaviors). After controlling for baseline differences, two comparison HR-ASD groups were included: 15 HR-ASD toddlers receiving behavioral intervention for 3 h per week for 5 months (INT+) and 15 HR-ASD toddlers who were only clinically monitored from age 18 months (INT−). Changes in social communication, restricted/repetitive behaviors, and language were assessed using standardized measures at pre- (T0) and post-intervention (T1). From T0 to T1, the INT+ group showed significant improvements in communication, social interaction, and language compared to INT− group. There was no effect on restricted/repetitive behaviors. Our findings highlighted the importance of early detection/intervention in autism and supported a positive impact of targeted interventions to improve outcomes in at-risk children.

## 1. Introduction

The prevalence of autism spectrum disorder (ASD) has shown a marked increase in the last decades. In Italy, a recent study of the National Institute of Health estimated a prevalence rate of 1 every 77 children in the 7–9 age range [1]. Symptoms of ASD occur early in life, with an 82% stability of ASD diagnosis by the age of 24 months [2]; however, diagnosis is typically not made before the age of 3 years. Early detection of ASD signs is a formidable challenge and an important step to early intervention, providing good opportunity for developmental benefits by taking advantage of early brain plasticity. As the first 24 months of child development are characterized by rapid changes in cognitive, communication, and social skills, providing early intervention during this critical period has a cascading effect on later development [3]. Prospective and longitudinal studies of siblings of children with ASD (showing a 13-fold increased risk for ASD by age 3 years [4]) are well suited to track typical and atypical developmental trajectories [5,6,7,8] and offer important advantages in terms of early detection/intervention prior to the emergence of ASD symptoms [9]. Previous studies reported that infants at high risk for ASD (HR-ASD) who are later diagnosed with the disorder show atypical development in communication, social skills, language, and sensory responsiveness by the age of 18 months, so a prompt and early intervention may mitigate the full onset of ASD-related symptoms.

Previous research on early intervention for toddlers newly diagnosed with ASD showed that long-term and intensive intervention can make a big difference in terms of outcomes and decrease the level of social communication impairment [10,11,12,13]. Toddlers with ASD aged between 18 and 30 months who received the Early Start Denver Model (ESDM) intervention for 20 h per week showed better scores on cognitive and adaptive behavior and reduced severity of ASD diagnosis [12]. In another study [14], children with ASD aged 21 to 33 months were randomized to receive an interpersonal or non-interpersonal synchrony intervention for 10 h per week for 6 months. Children with an additional interpersonal synchrony curriculum showed better social skills, such as imitation paired with eye contact and initiation of joint attention.

Even if intensive early intervention has important effects on the prognosis of ASD (e.g., [15]), it is plausible that families are not be able to afford an intensive long-term intervention program, and difficulties in obtaining funds from healthcare agencies are among the most important issues reported. Therefore, low- or moderate-intensity intervention becomes crucial and might represent a sustainable option.

Over the past few years, studies reported promising effects for infant siblings of children with ASD also with moderate-to-low intensity and short-term interventions in the first 3 years of life [16,17]. Rogers and colleagues [13] demonstrated that 6- to 15-month-old infants (n = 7) who were symptomatic for ASD showed a decrease in symptoms at 3 years after a low-intensity intervention (consisting of 12 consecutive weekly 1-h sessions, based on the ESDM intervention). Furthermore, a randomized controlled trial (RCT) [16] compared 9- to 11-month-old infants with a higher likelihood to develop ASD who received a low-intensity parent-mediated intervention with infants who were only clinically monitored from age 6 to 18 months. Infants receiving early intervention showed improvements in social attention, measured by EEG, and behavioral measures at age 12 and 18 months.

Another RCT study [18] examined the effect of a targeted social communication intervention (testing the adapted Video Interaction to Promote Positive Parenting) in a sample of 54 infants at high familial risk (from age 8 to 14 months). The results showed a moderate effect of the intervention on ASD-risk behaviors, attention skills, adaptive functions, and parental non-directiveness. No effects were found on language and brain measures (auditory ERPs).

Studies on the efficacy of different intervention strategies are available, but no standard approach is unanimously accepted and demonstrated, supporting the importance of an individualized intervention [19]. In early life, children with clinical signs of ASD show deficits in social communication skills, including difficulties in initiating of joint attention (pointing to share, eye-gaze shifting between objects and parents) and imitation behaviors [20]. In typically developing children, the ability to imitate, initiate, or respond to bids for joint attention is a developmental milestone that appears between the ages of 8 and 15 months [21]. Previous studies demonstrated that poor imitation skills and a lack of joint attention are discriminative characteristics in siblings of children with ASD and in children with early clinical signs of ASD [22,23,24,25]. These skills may be pivotal and are well recognized as predictors of later social and communicative development. Previous literature demonstrated that early intervention for these pivotal social skills may lead to behavioral changes/skill acquisition as well as normalized brain functioning [26] and may prevent potential secondary symptoms, such as emotional impairment and aggression [27]. Extensive empirical research supported the effectiveness of intervention based on the principles of Applied Behavior Analysis (ABA). ABA interventions can effectively reduce deficits in social skills and the most evident benefits of ABA practices are likely to occur in the first years of life [28].

Based on this overview, the present proof-of-concept study intends to contribute to the intervention literature by testing the effects of a pilot intervention in 18-month-old siblings of children with ASD who show emerging symptoms of ASD. The intervention focused on improving two of the pivotal skills of social communication—imitation and joint attention behaviors—by applying evidence-based behavioral strategies. We hypothesize that early intervention could have an effect on ASD-related traits, improving developmental outcomes in children who received early intervention compared to children with similar amounts of symptoms under clinical monitoring only, both in key social communication domain and in broader developmental skills, such as language and cognition.

A significant challenge of this study is to assess the effects of a short-term and lower-intensity behavioral intervention in 18-month-old Italian toddlers at familial risk for ASD, representing a unique sample and opportunity to improve our understanding of early detection and intervention in the prodromal developmental period prior to a potential diagnosis of ASD.

## 2. Materials and Methods

### 2.1. Study Design and Participants

A quasi-experimental design was applied to evaluate the efficacy of early intervention. Participants were recruited within an on-going longitudinal project aiming at identifying early risk markers for ASD [29]. Recruitment of HR-ASD was made possible through collaboration with the Italian Network for Early Detection of Autism Spectrum Disorders (NIDA Network).

We examined changes in cognitive, social communication, restricted/repetitive behaviors, and language in a group of 18-month-old toddlers at risk for ASD at pre-intervention (T0) and post-intervention (T1). The intervention group (INT+) was recruited between March 2018 and February 2020. Changes were compared to those of a control group of 18-month-old toddlers at risk for developing ASD (INT− group) monitored before (T0) and after (T1) a period of time of the same length as the intervention and recruited between March 2016 and February 2018. During this period, the experimental intervention protocol was not available. At T0, INT− toddlers with early clinical signs of ASD received clinical evaluation and, if needed, were included in a waiting list to receive treatment as usual. For the purpose of this study, we selected children in the INT− group without any intervention or specific activities aimed at improving social communication skills between T0 and T1.

Specifically, eligible criteria for the initial enrollment were: (1) having scores on the ADOS Calibrated Severity Scores of 3 or higher at initial assessment; (2) gestational age ≥ 36 weeks; (3) birth weight ≥ 2500 g; (4) Griffiths developmental quotient [30] > 70; (5) no major complications in pregnancy and/or delivery likely to affect brain development; and (6) absence of neurological deficits, dysmorphic markers, or other medical conditions.

**Figure 1:**
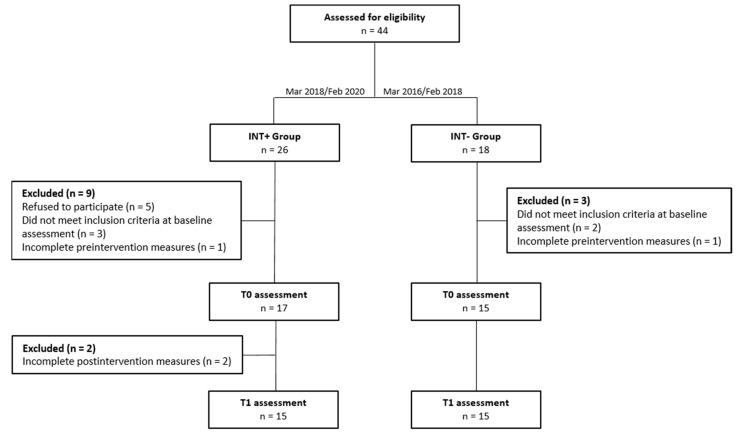
Participant flowchart.

Following inclusion criteria, 15 toddlers were included in the INT+ group and 15 toddlers in the INT− group (see Figure 1 for participant flowchart).

Written informed consent was obtained from all parents prior to testing. The study was conducted in accordance with the Declaration of Helsinki, and the protocol was approved by the Ethic and Scientific Committees of Eugenio Medea Scientific Institute (“5 per mille” funds for biomedical research, id. 752; id. 540; id. 845).

### 2.2. Materials

The examiners who administered the battery of standardized tests selected to carry out the comparison before and after the intervention were not those who directly conducted the intervention but rather external operators.

#### 2.2.1. Autism Diagnostic Observation Schedule—Second Edition (ADOS-2)

The Autism Diagnostic Observation Schedule—Second Edition (ADOS-2) is a semi-structured assessment of communication, social interaction, and restricted/repetitive behaviors for individuals suspected of having ASD [31]. The ADOS-2 includes five modules depending on different developmental, age, and language levels.

In this study, we used the ADOS Toddler module [32] to evaluate the severity of ASD symptoms at pre- and post-intervention. Two separate algorithms were applied based on age and language level: one for all children aged 12–20 months and nonverbal children aged 21–30 months and an algorithm for verbal children aged 21–30 months. We used Calibrated Severity scores [8,33] for the total ADOS score and for subdomains (Social Affect—SA and Restricted and Repetitive Behaviors—RRB) to provide quantitative estimates of ASD symptom severity, controlling for participants’ age and language levels [33].

#### 2.2.2. Griffiths Mental Development Scales—Extended Revised 0–2 (GMDS-ER)

The Griffiths Mental Development Scales—Extended Revised 0–2 (GMDS-ER) [30] provide a measure of development in children aged 0–2 years in 5 different domains (Locomotor, Personal-Social, Language, Eye and Hand Coordination, and Performance). For the purpose of the current study, the IQ scores (Mean of 100 and SD of 15) of each of the 5 different subscales at T0 and T1 were entered in the analyses. General Quotient (GQ) scores were used as inclusion criteria to exclude subjects with potential cognitive deficits (children with GQ ≤ 70 were excluded from the analyses).

#### 2.2.3. Child Behavior Checklist for Ages 1.5–5

The CBCL 1.5–5 is a 99-item, parent-report measure designed to record emotional and behavioral problems in toddlers; Italian adaptation by Frigerio et al. [34]. Each item describes a specific behavior and is scored on a 3-point Likert scale (0 = not true; 1 = sometimes true; 2 = very true). The scoring produces seven Syndrome Scales (Emotionally Reactive; Anxious/Depressed; Somatic Complaints; Withdrawn; Sleep Problems; Attention Problems; Aggressive Behavior) clustered in a summary profile made by Composite Scales (including Internalizing, Externalizing, and Total Problems scores) and five scales related to DSM-IV disorders (Affective Problems, Anxiety Problems, Pervasive Developmental Problems, Attention Deficit/Hyperactivity Problems, and Oppositional Defiant Problems).

In this study, we focused on the Withdrawn Syndrome scale and the Pervasive Developmental Problems (PDP) scale, based on previous literature showing good sensitivity and specificity in differentiating ASD children and controls [35,36]. Thus, Withdrawn and PDP T-scores (mean = 50; SD = 10) were entered in the analyses, and higher scores indicate greater problems.

#### 2.2.4. Language Assessment: Primo Vocabolario del Bambino

The Primo Vocabolario del Bambino (PVB) questionnaire is the Italian version of the MacArthurBates Communicative Development Inventory [37]—a parental questionnaire used to evaluate the expressive vocabulary size in young children. It is a checklist of 670 words, divided into 23 categories, including nouns (animals, vehicles, toys, food and drink, clothing, body parts, small household items, furniture and rooms, outside things, places to go), predicates (verbs and adjectives), function words (pronouns, question words, prepositions and locations, quantifiers and articles, connecting words), adverbs (words about time and location), sound effects and animal sounds, people, games and routines, and modal verbs. Parents were asked to mark the words produced and combined in sentences by their children [38]. At T0 and T1, the percentile scores of produced words were entered in the analyses to evaluate potential changes in expressive vocabulary size as a function of the intervention.

### 2.3. Intervention Procedure

Intervention sessions were conducted by licensed therapists with a training in applied behavior analysis (ABA) or behavior analysts, with the third (clinical psychologist) and last authors (child neuropsychiatrist) acting as coordinators of the intervention. One-to-one interventions for 3 h/week (1.5 h twice a week) were carried out over a period of 5 months (a total of about 40 sessions).

The behavioral intervention program focused on two target symptoms, imitation and joint attention behaviors, as two of the pivotal skills in early development that are positively associated with later development [39,40,41,42,43]. Behavior analytic techniques were used, including discrete trial, shaping for positive reinforcement, systematic prompting and fading procedures, and reinforcement procedures, according to the published manual [44].

The first sessions were used (a) to assess the child’s preferences according to Potential Reinforcer Profile developed by Amy McGinnis, M.S., OTR [45] to choose toys to be used in the intervention based on individual child’s preferences and interests (session 1) and (b) to establish baseline profiles of joint attention and imitation behaviors (sessions 2, 3, and 4). Baseline sessions consisted of ten opportunities for each child for each skill without prompting or reinforcing responses. Interventions for imitation and joint attention skills that did not reach the 80% mastery criterion were introduced.

The following sessions included imitation (about 45 min) and joint attention training (about 45 min). Imitation sessions started with imitation recognition and imitation of familiar actions (gestural and object imitation) and ended with the imitation of novel actions [46]. Joint attention evolved from initiating requests (starting with gaze shift with the child looking away from an interesting object to the adult and back) to coordination of gaze shift, vocalization and gestures through reaching, and pointing and showing behaviors [47]. Each session gradually increased in difficulty, based on the child’s spontaneous development. Parents regularly attended all intervention sessions. The intervention provided them with information and modeling to implement intervention strategies with their child, but no skills training was envisaged. Clinical monitoring visits by the last author took place at pre- and post-intervention.

### 2.4. Statistical Analyses

A series of repeated measures ANOVA with 2 (within-subject factor: Time, 2 levels: T0, T1) × 2 (between-subject factor: group, 2 levels: INT+, INT−) were conducted to examine cognitive, social communication skills, restricted/repetitive behaviors, and language over time as a function of intervention (significant interaction of Time × Group). Significant interactions were further explored by means of paired *t*-tests by comparing the two time points (T0 vs. T1) in each group. For each set of scales, the significance alpha threshold was adjusted to account for multiple testing (Griffiths Scales: 0.05/5 = 0.01; ADOS Calibrated Severity Scores: 0.05/3 = 0.02; CBCL Scales: 0.05/2 = 0.025; PVB questionnaire: 0.05/1 = 0.05).

There were no missing data for ADOS, CBCL, and PVB instruments both at T0 (pre-intervention) and at T1 (post-intervention, after 5 months). Data from three participants in the intervention group were missing on the Griffiths scales at T1. Statistical analyses were carried out using SPSS Statistics 25 software.

## 3. Results

### 3.1. Baseline Comparison

Descriptive statistics of individual, demographic, and clinical characteristics at T0 are shown in Table 1. *t*-Tests and chi-square statistics were used to compare the distribution of collected variables between INT+ and INT− groups. The two groups did not differ in terms of severity of ADOS symptoms at T0, sex, age, gestational weeks, and socioeconomic status (SES).

### 3.2. Group Comparison on Social Communication Skills and Restricted/Repetitive Behaviors

**Figure 2:**
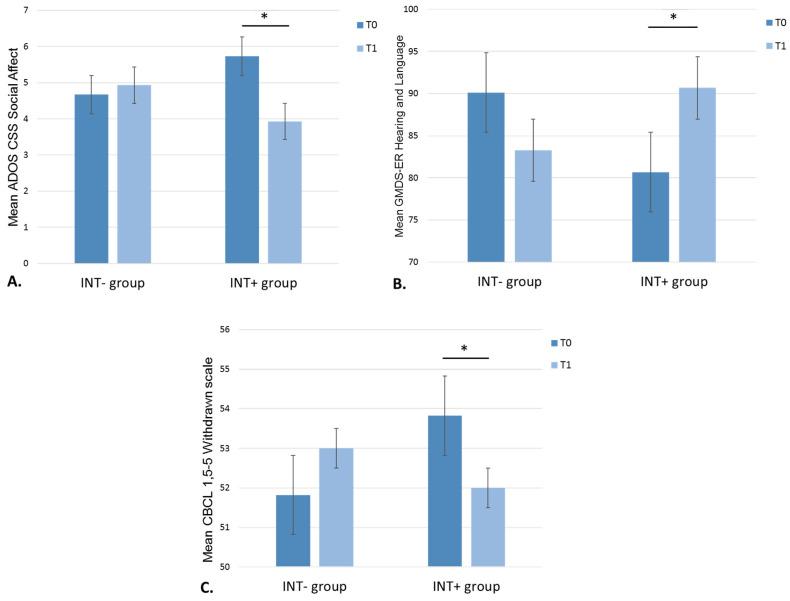
Graphical representation of mean standardized scores for ADOS CSS Social Affect (**A**), GMDS-ER Hearing and Language (**B**), and CBCL Withdrawn scale (**C**) at T0 and T1 in the two groups. Legend. Error bars show the 95% CI around each mean. Asterisks (*) indicate significant differences. INT−, group without intervention; INT+, group with intervention; CSS, Calibrated Severity Scores; GMDS-ER, Griffiths Mental Development Scales—Extended Revised 0–2; CBCL, Child Behavior Checklist.

There was a significant interaction effect of Time × Group on the ADOS CSS Social Affect (F(1,28) = 7.156; *p* = 0.012; partial η^2^ = 0.204; observed power = 0.733). Paired *t*-test contrasting ADOS CSS Social Affect scores at T0 vs. T1 in each group showed that the INT+ group had a significant decrease in the ADOS Social Affect scores between T0 and T1 (*t* = 3.473; *p* = 0.004) compared to INT− group (*t* = −0.465; *p* = 0.649) (see Figure 2A, Table 2). No significant main or interaction effects were found for ADOS CSS Restricted/Repetitive Behaviors. Considering ADOS CSS total scores, we found a trend towards significance for the interaction effect of Time × Group (F(1,28) = 2.968; *p* = 0.096).

### 3.3. Group Comparison on Cognitive and Language Skills

The repeated-measures ANOVA showed a significant interaction effect of Time × Group on the Griffiths Hearing and Language scale (F(1,25) = 7.900; *p* = 0.009; partial η^2^ = 0.240; observed power = 0.771). Paired *t*-test contrasting Griffiths scores at T0 vs. T1 in each group showed that the INT+ group had a significant increase in Hearing and Language scores between T0 and T1 (*t* = −2.272; *p* = 0.043) compared to INT− group (*t* = 1.839; *p* = 0.087) (see Figure 2B, Table 2). No significant main or interaction effects were found for other Griffiths measures. We found no significant main or interaction effects on the PVB questionnaire (F(1,28) = 1.704; *p* = 0.205). 

### 3.4. Group Comparison on Behavioral Profiles

The repeated-measures ANOVA showed a significant interaction effect of Time × Group on the Withdrawn Syndrome Scale (F(1,28) = 8.300; *p* = 0.010; partial η^2^ = 0.293; observed power = 0.782). Parents of children in the INT+ group reported a significant decrease in withdrawn problems over the course of the intervention (*t* = 2.285; *p* = 0.045). No significant changes were found in the INT− group (*t* = −1.759; *p* = 0.109) (see Figure 2C, Table 2). The Time × Group interaction on the Pervasive scale did not survive correction for multiple comparisons (F(1,28) = 4.410; *p* = 0.049).

## 4. Discussion

The present study evaluated the effect of early intervention in siblings of children with ASD with at-risk symptoms at the age of 18 months. We found that early intervention in siblings of children with ASD resulted in reduced core symptoms and greater developmental gains (language skills) compared to siblings with similar amounts of ASD symptoms who did not receive the intervention. Findings were in line with our expectations, and an intervention targeted on specific ASD features (i.e., imitation and joint attention) would specifically enhance social communication and language skills. For children until the age of 3 years, an updated review [23] supported the importance of interventions focused on specific key symptoms of ASD (e.g., lack of joint attention and emotional reciprocity, poor imitation) using an evidence-based approach, such as a behavioral and developmental approach.

Furthermore, even if an intensive and long-term treatment is still recommended [50], our findings add to intervention research by supporting possible improvements of ASD-specific deficits with short-term, lower-intensity, focused intervention for toddlers with clinical signs or a diagnosis of ASD [23]. In a sample of 38 toddlers with ASD, a previous study [40] aimed to examine if an 8-week intervention targeting joint attention behavior would result in improvements in social skills. The results showed significant gains in social skills (i.e., joint attention and type of functional play acts), and this effect was maintained at the 1-year follow-up. Another study [51] demonstrated a significant effect of 3 h per week for a 10-week targeted imitation intervention (i.e., Reciprocal Imitation Training) in a sample of preschool-aged children with ASD. Children who followed intervention showed better social skills (i.e., spontaneous imitation and numbers of play actions) compared to the control group at post-treatment. Taken together, our results are consistent with previous research demonstrating that toddlers with stronger joint attention skills can understand and respond to others’ intentions, more easily learn play actions, and then have better social skills. At the same time, children with better imitation behaviors are better able to imitate appropriate social interactions or to learn new ways to play with others. Most importantly, our results showing significant social gains in a relatively short period of time (5 months) are promising for toddlers at risk for ASD, as early intervention is crucial for better outcomes [26,27].

Our results showed that INT+ toddlers significantly improved on language compared to INT− group, as measured by the Griffiths Hearing and Language Scale, corresponding to wider repertoire of both receptive (basic concepts, general linguistic knowledge, verbal and semantic reasoning) and expressive language skills. This is consistent with previous literature showing that a short-term and focused intervention can lead to better language scores 12 months later [52]. In particular, it has been suggested that children with stronger joint attention and imitation skills similarly demonstrate greater language abilities (i.e., [53,54]), and these two skills are predictive of later language development in typically developing children [55]. It is well-known that joint attention has an important role on language because language is learned also through episodes of joint attention (for example, a child could learn object label during joint attention episode, improving the receptive vocabulary repertoire). Moreover, by copying motor and language skills, children improve communication with the world around them [53]. We may speculate that our findings demonstrate the effects of this pilot intervention to increase social skills but also outcomes more distal to the intervention targets (such as language). It is noteworthy, however, that change in expressive vocabulary (i.e., number of words that the child spontaneously produces), as measured by the Italian version of the MacArthur-Bates Communicative Development Inventory, was not different between the two groups. We could not identify individual characteristics of children who showed greater improvement in expressive skills owing to the small numbers of toddlers in each group, and research in larger groups is needed to test individual differences in vocabulary growth. Further replication studies should confirm this result, especially because most of the previous studies have focused more generally on language as a whole rather than receptive versus expressive language [56].

To further support our findings, we found significant reductions in parent-reported withdrawn symptoms for children in the INT+ group. Intervention sessions may help parents to observe and become aware of child difficulties. Furthermore, parent reports of significantly improved behavior could demonstrate generalization of intervention gains across different settings (e.g., home setting). However, these are speculations that need to be further studied before drawing any conclusions.

Finally, our results did not show group differences on restricted and repetitive behaviors (RRBs). A possible explanation for this may be related to the baseline characteristics of our sample. The two groups did not show significant numbers of RRBs at T0, as measured by ADOS, and it is plausible that statistical power and variability were not enough to detect potential group differences. However, since RRBs are detectable by age 12 months, become more evident at age 24 months [57], and represent one of the primary stressors for caregivers [58], future intervention research should examine this effect in larger and more representative samples. If these data are replicated and confirmed, they may be useful to track novel interventions given the paucity of intervention practices focused on RRBs in ASD [59].

This proof-of-concept study presents several limitations. First, this is not a randomized controlled trial study, which limits the possibility to demonstrate a causal association between the intervention and outcomes. Second, follow-up measures are not available to examine maintenance of intervention effects. Unfortunately, owing to COVID-19 restrictions, we could not administer short-term follow-up measures to most of the children involved in this study. A longer follow-up would be required to assess long-term intervention effects.

Besides these limitations, the strengths of this pilot study need to be acknowledged. Although a prospective quasi-experimental study is less rigorous than a RCT study, INT+ and INT− groups were well characterized and matched for clinical (amounts of ASD symptoms), individual, and sociodemographic variables at the baseline. Furthermore, inclusion of children at increased risk of ASD in early intervention studies is a challenge since enrollment of this at-risk population has been found to be difficult, and research studies are still scarce [60]. Thus, the present study may be promising given the growing need for early intervention in the prodromal developmental period prior to a diagnosis of ASD.

## 5. Conclusions

As a concluding remark, even if these findings need replication in a larger and randomized controlled study, including long-term follow-up measures, they seem to suggest that early intervention over a 5-month period can be effective for improving outcomes of toddlers at risk for ASD, with beneficial effects not only on key domains symptoms but also on broader developmental skills. If these results are confirmed, the current pilot intervention may be not only effective but also cost efficient. Future research focused on the potential neurocognitive biomarkers that moderate and predict early intervention response is of great interest, especially because there is a high heterogeneity in how individuals respond to intervention [15].

## Figures and Tables

**Table 1:** Baseline characteristics for INT+ and INT− groups.

		INT+ Group (n = 15) Mean (SD)	INT− Group (n = 15) Mean (SD)	*t*-Test *p*-Value
**Age (months)**		19.26 (1.12)	18.69 (0.82)	0.123
**Gestational age (weeks)**		39.38 (1.32)	39.23 (.83)	0.726
**Birthweight (g)**		3299.23 (441.50)	3422.14 (450.70)	0.481
**Socioeconomic status (SES) ^a^**		48.57 (16.10)	56.43 (19.46)	0.255
**ADOS Calibrated Severity Scores**	Social Affect	5.73 (2.66)	4.67 (1.39)	0.180
	Restricted/Repetitive Behaviors	4.53 (1.92)	3.80 (2.46)	0.370
	Total score	5.13 (2.36)	4.47 (1.60)	0.372
**Griffiths Mental Development Scales**	Locomotor ^b^	106.21 (14.14)	103.33 (9.75)	0.526
	Personal-Social ^b^	94.57 (15.18)	91.53 (17.55)	0.623
	Hearing and Language ^b^	79.57 (14.02)	90.13 (12.53)	0.072
	Eye and Hand Coordination ^b^	97.50 (14.02)	100.73 (13.68)	0.535
	Performance ^b^	96.57 (16.83)	100.13 (18.39)	0.590
**PVB questionnaire ^c^**	Expressive Vocabulary ^d^	22.33 (22.90)	25.00 (25.66)	0.774
**Child Behavior Checklist 1.5–5**	Withdrawn ^e^	53.07 (4.36)	51.54 (4.67)	0.386
	Pervasive Developmental Problems ^e^	54.64 (6.80)	51.46 (4.39)	0.165

^a^ Socioeconomic status (SES) was scored according to Hollingshead 9-point scale [48], whereby a score ranging from 10 to 90 was assigned to each parental job, and the higher of two scores was considered when both parents were employed; ^b^ Age-standardized IQ scores (M = 100; SD = 15) in Griffiths Mental Development Scales—Extended Revised [30]; ^c^ PVB, Primo Vocabolario del Bambino [38]; ^d^ Percentile scores; ^e^ T-scores (M = 50; SD = 10).

**Table 2:** Descriptive statistics of the two groups (INT+, INT−) at T0 and T1.

		INT+ Group		INT− Group		
		T0	T1	T0	T1	Significant Post-Hoc Analyses
		Mean (SD)	Mean (SD)	Mean (SD)	Mean (SD)	
**ADOS Calibrated Severity Scores ^a^**	Social Affect	5.73 (2.66)	3.93 (2.31)	4.67 (1.40)	4.93 (2.74)	INT + (T0) > INT + (T1)
	Restricted/Repetitive Behaviors	4.53 (1.92)	3.93 (2.28)	3.80 (2.46)	3.60 (2.26)	
	Total score	5.13 (2.36)	3.67 (2.16)	4.47 (1.60)	4.40 (2.64)	
**Griffiths Mental Development Scales—Extended Revised 0–2**	Locomotor ^b^	104.67 (12.21)	114.00 (11.30)	103.33 (9.75)	108.80 (15.88)	
	Personal-Social ^b^	96.33 (15.62)	97.92 (15.45)	91.53 (17.55)	93.80 (17.19)	
	Hearing and Language ^b^	80.67 (16.89)	90.67 (18.07)	90.13 (12.53)	83.27 (21.89)	INT + (T0) < INT + (T1)
	Eye and Hand Coordination ^b^	99.08 (13.67)	98.50 (12.86)	100.73 (13.68)	98.73 (9.84)	
	Performance ^b^	99.25 (16.42)	99.17 (14.20)	100.13 (18.39)	102.00 (12.26)	
**PVB questionnaire ^c^**	Expressive Vocabulary ^d^	22.25 (23.60)	20.42 (19.24)	25.00 (25.66)	13.85 (13.72)	
**Child Behavior Checklist 1.5–5**	Withdrawn ^e^	53.82 (4.66)	52.00 (3.80)	51.82 (5.06)	53.00 (6.86)	INT + (T0) > INT + (T1)
	Pervasive Developmental Problems ^e^	55.36 (7.42)	52.00 (3.49)	51.73 (4.75)	52.55 (7.15)	

^a^ Calibrated Severity Scores based on Esler et al. [33]; ^b^ Age-standardized IQ scores (M = 100; SD = 15) in Griffiths Mental Development Scales—Extended Revised 0–2 [30]; ^c^ PVB, Primo Vocabolario del Bambino [38]; ^d^ Percentile scores; ^e^ Age-standardized T-scores (M = 50; SD = 10) in Child Behavior Checklist 1.5–5 [49].

## Data Availability

The data that support the findings of this study are available from the corresponding author, V.R., upon reasonable request.
